# Know-Me: A Toolkit for Designing Personalised Dementia Care

**DOI:** 10.3390/ijerph18115662

**Published:** 2021-05-25

**Authors:** Gubing Wang, Armagan Albayrak, Eef Hogervorst, Tischa J. M. van der Cammen

**Affiliations:** 1 Faculty of Industrial Design Engineering, Delft University of Technology, 2628 CE Delft, The Netherlands; a.albayrak@tudelft.nl (A.A.); t.j.m.vandercammen@tudelft.nl (T.J.M.v.d.C.); 2 School of Sport, Exercise and Health Sciences, Loughborough University, Loughborough LE11 3TU, UK; E.Hogervorst@lboro.ac.uk

**Keywords:** dementia, human-centred design, design tool, personalised dementia care, ergonomics, co-design, data-enabled design, design education, nursing homes

## Abstract

Personalisation is a crucial element in providing person-centred care for people with dementia. This paper presents the development and evaluation of a design toolkit to facilitate the work of designers and healthcare professionals in personalising dementia care. This toolkit, named “Know-me”, was grounded in the findings of Ergonomics in Aging, Co-design, and Data-enabled Design, derived from literature review and from the field during a four-year doctorate project. “Know-me” was designed to be easily accessible, flexible, and engaging, providing concrete and hands-on guidance for designers and healthcare professionals to use in designing for personalised dementia care. A proof-of-concept evaluation of the “Know-me” toolkit was conducted via student projects on design for dementia care. During this process, we found that “Know-me” could be adapted flexibly so that the care team could use some of the tools by themselves. A feature-by-feature comparison of the “Know-me” toolkit with similar state-of-the-art toolkits was conducted, and based upon this, the strengths and weaknesses of the “Know-me” toolkit are discussed. This preliminary study indicates that the “Know-me” toolkit is a helpful addition to the current pool of toolkits on designing for dementia care.

## 1. Introduction

The World Health Organisation (WHO) predicts that the number of people living with dementia worldwide will increase to 132 million by 2050 [1]. Dementia is a syndrome (a group of related symptoms) associated with an ongoing decline of brain functioning, usually of a chronic or progressive nature, in which there is a disturbance of multiple higher cortical functions, including memory, thinking, orientation, comprehension, calculation, learning capacity, language and judgement affecting activities of daily life [2]. These symptoms lead to an increased burden of care and increased costs for governments, communities, families and individuals, and a loss in productivity for economies [3].

In response, WHO initiated a global action plan on supporting the public response to dementia spanning from 2017 to 2050, with the aim to improve the physical, mental and social wellbeing of People with Dementia (PwD), their caregivers and families [1]. This plan highlighted that the current gap between the care needed by PwD and the care provided to them is wide [1]. As dementia progresses, impairments of cognitive function are commonly accompanied and occasionally preceded by deterioration in emotional control, social behaviour, or motivation [2], which add extra challenge and burden to dementia care. Around the 1990s, there was a movement in the field of dementia care, suggesting that PwD should be cared for in a person-centred manner. The “person-centred” perspective on dementia care was introduced in the care practice by Kitwood [4], where “the personality, past experiences, health, and other aspects of the person with dementia also influence how the person will behave in addition to neurological impairments.” Hence, neurological impairment is important but is not the only component to be considered when caring for PwD. Each person with dementia is different, PwD do not lose their individuality, and therefore, as far as possible, PwD should be treated in an individualised way, taking into account their personality, life experiences and preferences. Therefore, personalisation is the core of Person-Centred Care.

### 1.1. Related Work

In the healthcare field, the tools for facilitating care personalisation are becoming increasingly data-driven [5]. These tools are mainly digital and often applied in chronic condition management, such as cancer [6], physiotherapy [7], cardiovascular disease [8] and elderly care [9]. The patients are usually required to be cognitively able to set personalised goals and reflect on the data together with healthcare professionals. However, people in moderate to late stages of dementia usually have difficulties with goal setting and communication in general. Healthcare professionals in dementia care are currently mainly facilitated by training courses [10] and protocols [11] for delivering personalised care.

In the design field, an increasing number of design tools have been created for dementia. For example, Loughborough University developed “persona” [12] as a tool to facilitate architects in designing homes and spaces for PwD. This model did not focus on care but was developed to provide insights in the various stages of dementia and the changes in needs of PwD, as well as the associated design needs between them and their environment. Systematic toolkits for designing for dementia care are limited. To the authors’ knowledge, there are currently two projects that have developed toolkits for designing for dementia care, i.e., LAUGH [13] and MinD [14].

The toolkit developed by MinD focuses on design for PwD as a user group. Some tools are more focused on how to involve PwD in the design process [15,16], while others are guiding designers to consider the physical capabilities and psychological and social needs of PwD [17]. The Compassionate Design Toolkit by LAUGH is the first and only toolkit so far to guide designers in creating personalised designs for PwD, which is focused on the senses and life history of PwD [18]. However, personalised designs have long been regarded as expensive and have low social impact. Thankfully, with the development of technology, personalised designs are becoming more prevalent, as exemplified by smart speakers, recommendation systems, fitness trackers and other products and services that can adapt to individual’s preferences, behaviours and experiences effortlessly [19]. These products and services point out a direction for the development of personalised designs for PwD to be more comprehensive, low-cost and hence, having a higher socioeconomic impact.

In this light, we developed a toolkit (named “Know-me”) to support designing for personalised dementia care. This toolkit was developed to further address the challenges faced by both healthcare professionals and professional designers. For healthcare professionals, the “Know-me” toolkit could be augmented with current training and protocols and hence, facilitate personalised care for PwD. For professional designers, the “Know-me” toolkit could introduce the data-driven direction for personalisation to help designers in designing personalised dementia care. In comparison to the toolkits by MinD and LAUGH, the “Know-me” toolkit adds the component of data-driven direction for personalisation and guides designers and healthcare professionals to work together for personalising dementia care. These features make the “Know-me” toolkit suitable to be applied in design scenarios where: both designers and healthcare professionals will be involved; sensor data can be collected from PwD under ethical considerations; the design outcome is to achieve personalised dementia care. We consider the “Know-me” toolkit as an addition to the toolkits developed by LAUGH and MinD.

### 1.2. The Know-Me Toolkit

**Figure 1:**
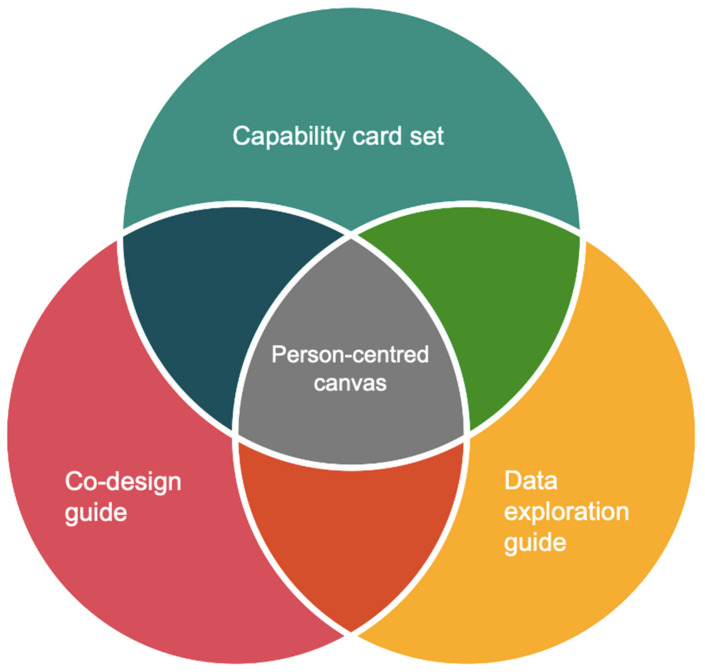
The relations between the four tools in the “Know-me” toolkit for personalising dementia care (i.e., Capability Card Set, Co-design Guide, Data Exploration Guide, Person-centred Canvas).

The “Know-me” toolkit consists of a user manual and four tools, which are a capability card set, a co-design guide, a data exploration guide, and a person-centred canvas. The tentative relations between these four tools are illustrated in Figure 1. Specifically, the insights generated by the capability card set, co-design guide and data exploration guide feed into each other, and together, they contribute to the person-centred canvas. The envisioned users of this toolkit are designers and healthcare professionals (referred to as “users” below); specifically, it is envisaged that designers will co-design with healthcare professionals using the “Know-me” toolkit to support designing for personalised dementia care.

Each tool of the “Know-me” toolkit will be introduced below. A website was developed to represent this toolkit, which played a central role in disseminating the toolkit to potential users. The readers can view further details of the toolkit via the website link below: www.designfordementia.squarespace.com (accessed on 20 May 2021).

#### 1.2.1. Capability Card Set

The aim of the capability card set is to help users to identify the remaining capabilities of PwD. The capability card set was created based on the exploration of the Ergonomics in Ageing approach in designing for personalised dementia care. The initial recommendation list was generated based on a literature review [20] and refined in a field study [21]. There are 11 cards in total, and each corresponds to a capability that is commonly different in older adults to younger adults. The capability cards were categorised according to their categories in ergonomics (i.e., cognition-, sensory-, and movement-oriented capabilities) and have their corresponding icons at the right bottom corner. On each card, a question is proposed to help users to identify possible capability limitations an older adult might have. Below the question, several suggestions are made about what could be done to allow one to still be able to interact with this older adult if they have certain capability limitations.

**Figure 2:**
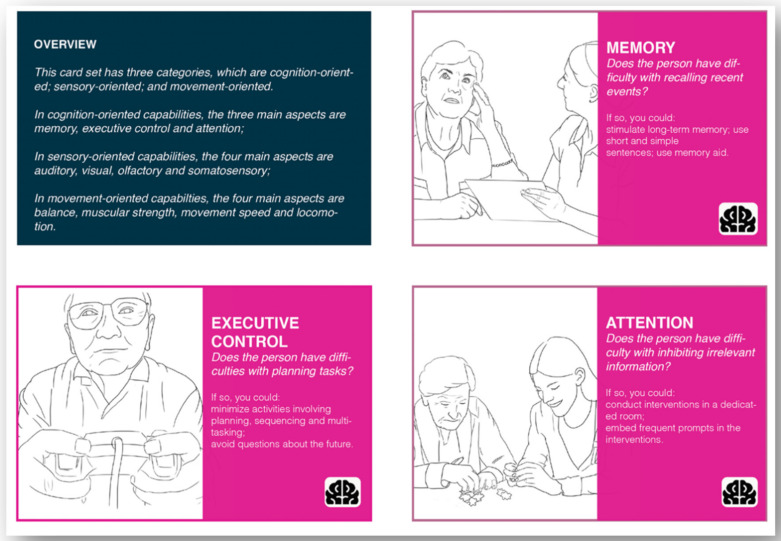
The overview instruction card and the cards for cognition-oriented capabilities.

**Figure 3:**
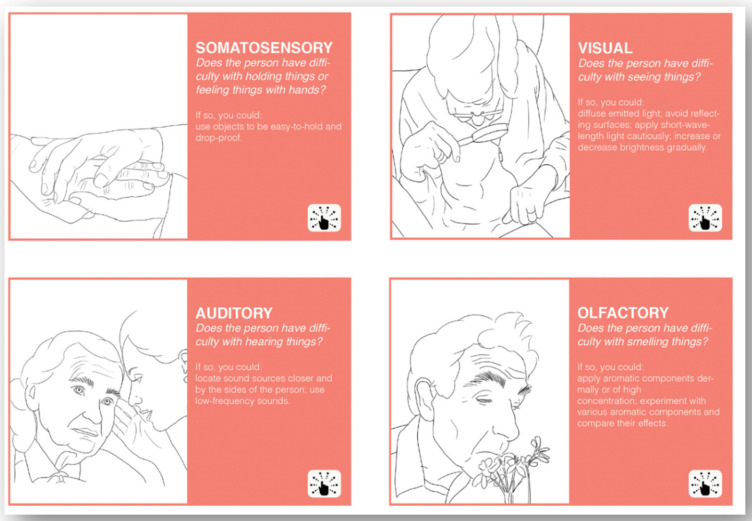
The cards for sensory-oriented capabilities.

**Figure 4:**
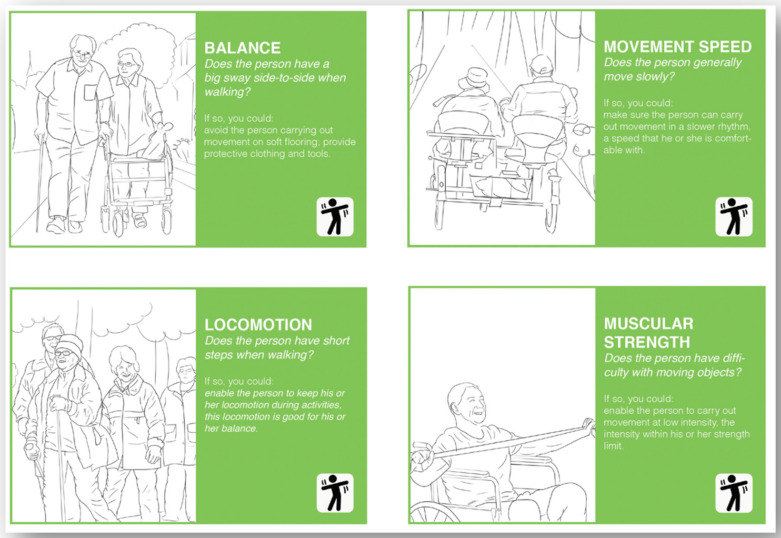
The cards for movement-oriented capabilities.

The overview instruction card and the cards for cognition-oriented capabilities can be found in Figure 2. The cards for sensory-oriented capabilities can be found in Figure 3. The cards for movement-oriented capabilities can be found in Figure 4. The full card set can be downloaded from the website www.designfordementia.squarespace.com (accessed on 20 May 2021).

By using this card set, users can explore what PwD can still do when their capability limitations are taken into account. Hence, it is based on capabilities rather than limitations and hence, implements a positive psychology approach. We advise the cards to be printed out so that they can be flexibly spread out, studied individually, placed together, and shared with other members if one is working within a team [22].

As mentioned above, the capability card set was generated based on a literature review [20] and a field study [21]. We realise that the list of capability considerations for PwD might be longer. For example, swallowing problems (dysphagia) may occur for some PwD. This aspect might be a useful addition to design toolkits for dementia care in the future.

#### 1.2.2. Co-Design Guide

**Figure 5:**
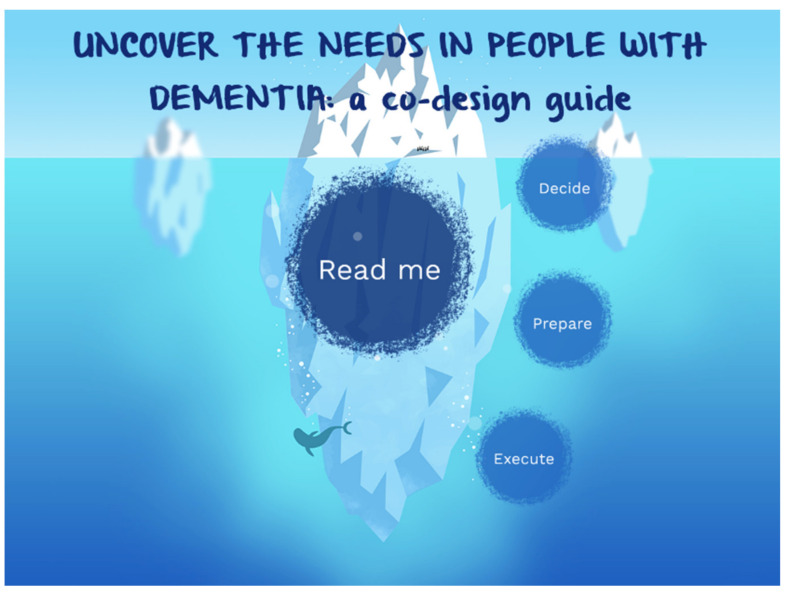
Home page of the co-design guide.

The goal of this guide is to help users to uncover the needs of PwD. The co-design guide is created based on the exploration of the Co-design approach in designing for personalised dementia care. The first part of the exploration has been done using a scoping review [23], the outcome of which has been evaluated in a field study [21]. The co-design guide is visualised digitally in a way that is easy to navigate (zoom-in, zoom-out). It is interactive with a layered design and provides an overview to make the information more digestible for the users. The home page of the co-design guide is shown in Figure 5.

On the home page, a “Read me” bubble is located at the centre to provide the background of the co-design guide. This guide is categorised into three bubbles, which are: “Decide”, “Prepare” and “Execute”, and they correspond to “decide on whether co-design is suitable”, “how to prepare a co-design session”, and “how to execute the co-design session”, respectively. The full guide can be accessed from the website www.designfordementia.squarespace.com (accessed on 20 May 2021).

#### 1.2.3. Data Exploration Guide

**Figure 6:**
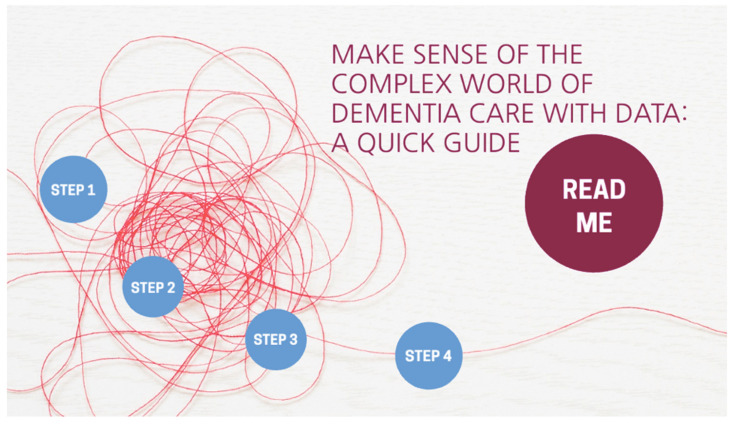
Home page of the data exploration guide.

The goal of this guide is to help the users to generate more insights in PwD by combining quantitative data and qualitative data. The data exploration guide is created from the exploration of the Data-enabled Design approach in designing for personalised dementia care. The concept of this guide was first formed and then established in two field studies, respectively [24,25]. This guide is visualised digitally in a way that is easy to navigate (zoom-in, zoom-out). It is interactive with a layered design and provides an overview to make the information more digestible for users. The home page of the data exploration guide is shown in Figure 6.

On the home page, a “Read me” section is located near the title to provide a background of this guide to the users. The guide is divided into four steps, which are “what data to be collected”, “the value of collected data”, “how to collect and visualise collected data”, and “how to facilitate data analysis”. Within each step, a description of the step is given and accompanied by some tips and more resources that the users can dig into. The full guide can be accessed from the website www.designfordementia.squarespace.com (accessed on 20 May 2021).

#### 1.2.4. Person-Centred Canvas

**Figure 7:**
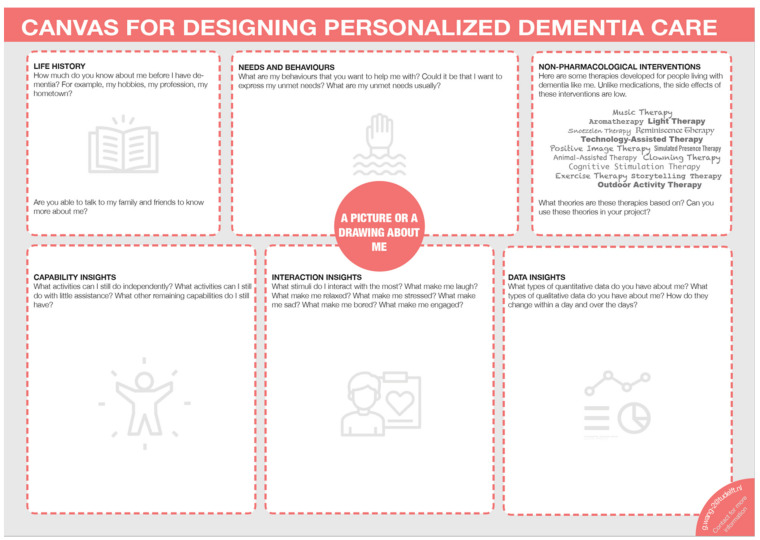
Template of the person-centred canvas.

The aim of this canvas is to help the users to have a clear overview of which aspects need to be considered when designing for PwD. The person-centred canvas is developed from the Need-driven Dementia-compromised Behaviour model (NDB model) [26], enriched by a systematic review on non-pharmacological interventions for dementia care [27], and established after investigation of the three design approaches (i.e., Ergonomics in Aging, Co-design, Data-enabled Design) as mentioned before. A template of the canvas is shown in Figure 7.

The canvas is intended to guide the users on what aspects of information to gather for designing personalised care; it also helps the users to map, manage, and keep track of all the information and insights they have gathered about a person with dementia in an integrative way. At the same time, this canvas is intended to inspire the users by presenting a summary of current non-pharmacological interventions for managing Behavioural and Psychological Symptoms of Dementia (BPSD). This is because the majority of PwD (97%) will develop at least one BPSD symptom as their dementia progresses [28]

The canvas is divided into six sections, which are “life history”, “needs and behaviours”, “non-pharmacological interventions”, “capability insights”, “interaction insights”, and “data insights”. Capability insights correspond to the findings of applying the capability card set; “life history” and “interaction insights” correspond to the discoveries of applying the co-design guide; “data insights” correspond to the insights gained by using the data exploration guide. At the centre of the canvas, users are encouraged to put a photo or a hand-drawing of the person with dementia. This method has been found to be helpful in stimulating person-centred empathy during design [29].

The envisioned connections of the six sections of the canvas are the following: the users are encouraged to involve caregivers, family members and friends of the person with dementia to collect past stories about the person (“life history”). This will help the users to zoom out from the current state of the person and see the person as a whole. Then the users are advised to frame the behaviours of the person as a way for expressing their unmet needs (“needs and behaviours”). This could be done through interviews with caregivers or careful observation of the person. To understand what the unmet needs could be, the users could combine the insights gathered about the current state of the person (“capability insights”, “interaction insights”, and “data insights”) and take into account the past of the person (“life history”) to form a comprehensive view of this person. In this way, all the factors suggested by the NDB model will be covered, which helps the users to identify the possible unmet needs. After the unmet needs are identified, the users can then refer to the interventions (“non-pharmacological interventions”) for inspiration; and use all the insights presented in the canvas to design for the person. The summary of non-pharmacological interventions is intended to help the users to be aware of what interventions are out there in the medical field and thus dig into each intervention if needed (instead of performing a literature review from scratch). The users do not have to stick to one type of intervention; rather, they are encouraged to think about the theories behind the proposed interventions and how these theories could be applied when designing personalised dementia care.

Ultimately, this canvas could also help the users share and reflect on the information and insights, which are the basis of the design, with important stakeholders in the design process. Applying this canvas in real-life settings could be an iterative process and not as linear as suggested, yet this envisioned connection gives a direction about how to use the canvas.

This canvas can be used in both a physical and digital format depending on the users’ preference. We recommend users write the information and insights on post-its (could be physical or digital) and then position the post-its in the appropriate sections of the canvas. The canvas offers a structure to organise post-its, and the post-its have the flexibility to be rearranged along the design process. The full-size canvas can be downloaded from the website www.designfordementia.squarespace.com (accessed on 20 May 2021).

#### 1.2.5. User Manual

We encourage the users to explore their own ways of using the toolkit. However, a user manual is written to guide users who would like to have a structured approach in using these tools in the design process. The double diamond model [30]—the most-cited design process model—is used as a reference to inspire the designer about how these tools might fit in the design process. According to the double diamond model, the design process can be divided into four phases, which are: discover, define, develop, and deliver.

Specifically, in the Discover phase, all tools are involved; in the phases of Define and Develop, the person-centred canvas and the capability card set are involved; in the Deliver phase, the co-design guide and data exploration guide are involved. The contribution of each tool at the different design phases is slightly different, and their intended roles are outlined in the user manual. The full user manual can be found on the website www.designfordementia.squarespace.com (accessed on 20 May 2021).

To summarise, from using the “Know-me” toolkit, the designers and healthcare professionals could be guided to understand each person with dementia in multiple aspects. These aspects include and are not limited to: the remaining capabilities of the person; the psychosocial factors of the person (e.g., life history); the preferred interaction approaches of the person; the preferred stimuli of the person; the health status of the person; the physical and social environment that the person is usually in; and the physical and social environment that the person prefers. The “Know-me” toolkit provides a hands-on, systematic, and efficient way for designers and healthcare professionals to gain insights into these aspects for each person with dementia. Therefore, the “Know-me” toolkit contributes to the design for personalised dementia care by helping designers and healthcare professionals to develop a comprehensive understanding of each person with dementia.

### 1.3. Aims and Objectives

The Know-me toolkit was then evaluated with the following research questions:

1. What is the perceived usefulness of this toolkit?
2. What are the desired improvements for this toolkit?

To answer the research questions, the toolkit was evaluated by student projects focusing on dementia care design during the Advanced Concept Design course at the Faculty of Industrial Design Engineering (IDE), Delft University of Technology (TU Delft).

## 2. Materials and Methods

### 2.1. Context

Advanced Concept Design is a compulsory course for Masters students majoring in Integrated Product Design at the Faculty of IDE, TU Delft. This course focuses on the conceptualisation phase of product development and starts from a real-life design challenge and ends with the concept of a product or a product-service system. The design challenge was co-created with RespectZorg nursing home, a non-profit care organisation in the Netherlands. RespectZorg then teamed up with Vegro, a wholesale company of care aids in the Netherlands, to provide students with information and support during the assignment. The design challenge was related to improving dementia care in nursing homes, and the specific assignment was:

Design a product/product-service system in the nursing home that can engage people with dementia in meaningful activities (alone, with family members, or as a group) for 30 min a day.

In total, 16 students participated in this course. The students were divided into three subgroups with three sub assignments: (1) to reduce apathy during the morning coffee break; (2) to reduce restlessness in the afternoon; (3) to reduce wandering behaviours.

Students worked on the project for 20 weeks. In the first ten weeks, the students worked in subgroups to understand the design context and received guidance from experts in the field of culture, ergonomics, and technology. In the last ten weeks, each student came up with his/her own design direction, developed concepts and prototypes, and delivered an animation and report about their final designs.

Due to COVID-19, the students were not able to access the nursing home, which made the research phase especially difficult for them. Specifically, they received limited support from healthcare professionals and were not able to meet PwD in person. Some students were not able to test their design concepts in the nursing home, while other students sent their prototypes to the nursing home and tested them remotely. The principal researcher (GW) acted as an expert in the field of dementia care design and provided relevant information and insights to the students who reached out.

### 2.2. Procedure

GW introduced the toolkit to the students in Week 15 via an online lecture. After the toolkit was introduced, the students were able to ask questions about the toolkit in a personal Q&A with the author. All 16 students asked questions about the toolkit and other questions related to their designs. GW was also present in future meetings to give advice on how to use the toolkit and other questions related to dementia care design.

**Figure 8:**
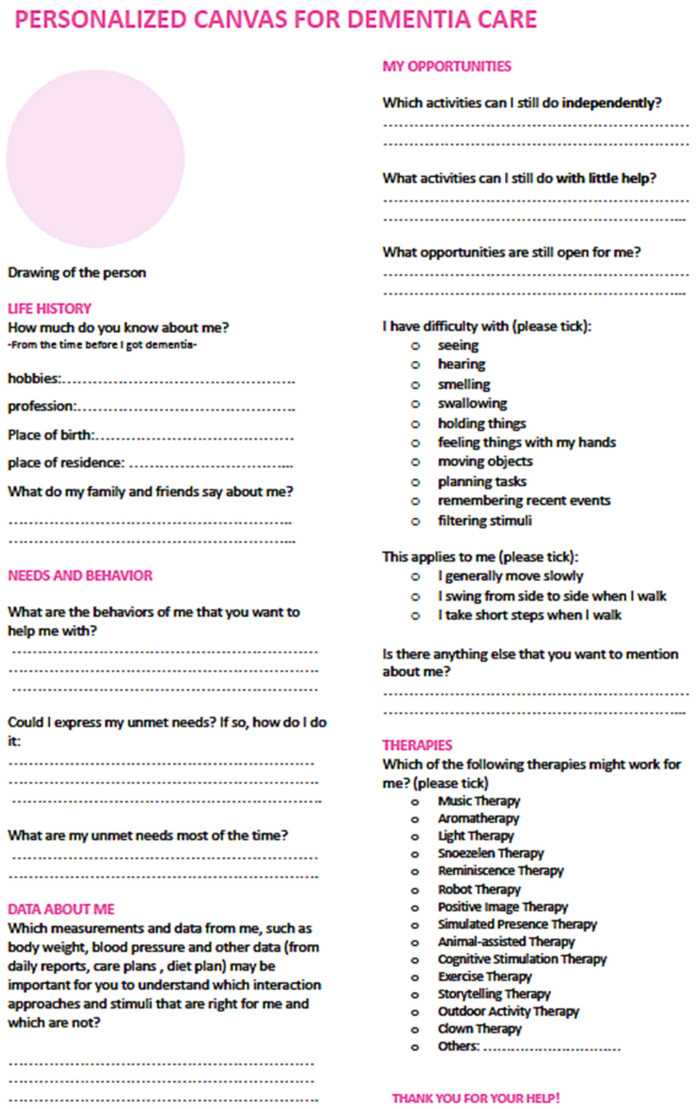
The person-centred canvas template for the care team (translated in English).

In Week 16, GW was approached by the course coordinator, who is a designer herself, suggesting the possibility of creating a care team version of the person-centred canvas. The course coordinator postulated that the care team could fill in the canvas by themselves with the information of the residents they are taking care of. In this way, the students could receive some real data about PwD that they were designing for without adding too much workload to the care team. Together with the course coordinator, a care team version of the person-centred canvas was created by adapting the original person-centred canvas. The person-centred canvas template of this care team version is shown in Figure 8.

The person-centred canvas was adapted to be more accessible to the care team in a few ways. First, the design terminologies were replaced with everyday language (e.g., “capability” is replaced with “what one can do”). Second, the layout was adapted from A3 to A4 since most of the offices in the nursing home can only print A4 papers. Third, the capabilities mentioned in the capability card set were added to the canvas to help the care team reflect on the remaining capabilities of each resident. Moreover, some short answer questions were changed into a checklist since the care team has limited time, and they are more familiar with a structured way of working. Lastly, the language changed from English to Dutch since this canvas was deployed in the Netherlands, where all caregiver participants are native Dutch speakers.

In Week 17, the care team filled in six person-centred canvases, each representing a resident in the nursing home. These canvases were sent to the students immediately via email. In addition to these meetings and materials, the students were free to choose other design methods and tools during the design process.

In Week 20, the students were asked to reflect on their design process and the Know-me toolkit via an online questionnaire. The questionnaire consists of open questions and multiple-choice questions with a single-choice option. In the questionnaire, six items were evaluated, which were: the user manual, the co-design guide, the data exploration guide, the capability card set, the original person-centred canvas and the filled-in canvas by the care team. Each student was asked to select the tools that they found the most and the least useful in the toolkit. If none of the options applied, the students could select “others” and write their answers. The students were then asked to give the reasons behind their choices by the open-ended questions. It was explicitly mentioned in the questionnaire that the answers would not be taken into account during grading, and the author was not involved in the grading process to allay concerns about social desirability bias.

The author (GW) then conducted a thematic analysis of the collected data together with a student assistant. Following the guidance of Braun and Clarke [31], they got familiarised with the data independently first, and three discussion sessions were conducted for generating initial codes and searching for themes, reviewing themes and defining themes until consensus was reached. All discussions were facilitated by a Miro board.

## 3. Results

Ten out of 16 students responded to the questionnaire. Only one participant had previous experience with designing for PwD. Five students rated their satisfaction with their designs at six, and five other students rated at five on a seven-point Likert scale (1: not satisfied; 7: very satisfied). All students indicated that they would have appreciated direct interactions with PwD and more involvement of caregivers. The outcomes of the thematic analysis are summarised in Table 1 and Table 2, respectively. Table 1 sums up how the toolkit was perceived to contribute to the design process, while Table 2 encapsulates the desired improvements of the toolkit.

In Table 1, four themes were identified that the toolkit could help to “understand the context and users”, “initiate the ideation”, “develop the concept”, and “test the prototype”. To understand the context and users, the toolkit was perceived by the students to be able to provide knowledge about PwD before field study, as well as help them to gain insights from the care team and narrow down the design brief. The capability card set was mentioned by a few students to help them understand the capabilities of PwD. In terms of initiating the ideation process, the toolkit was reported to offer design directions and help students with understanding the needs and wishes of PwD. As for developing the concept, the toolkit was used as a knowledge source and could provide criteria for improving the design. Regarding prototype testing, some students found the toolkit helped them to answer user testing questions and to predict the reactions of PwD to their prototypes.

In Table 2, three themes were identified that the toolkit could be improved on, i.e., “elaborate the tools”, “add more elements to the toolkit”, and “make the toolkit more accessible”. Specifically, the students commented that the tools could be elaborated in three ways, which were: provide more examples about how each tool could be used; increase the details of the capability cards; include how the toolkit could be used with the care team. In addition, the students would like to see the toolkit to include three elements in addition to the current scope, which were: literature resources about dementia; methods for remote testing; methods for evaluating responses of PwD. As for the accessibility of the toolkit, the students indicated the toolkit would be more helpful if: the visibility of the website were higher in the Google search; the user manual was easier to find on the website; hard copies of the toolkit were given with templates; and the toolkit was provided at an earlier stage of the design process.

## 4. Discussion

### 4.1. Main Findings

This evaluation was aimed at identifying the perceived usefulness of the Know-me toolkit and desired improvements as obtained from designers. The toolkit was found to be able to contribute to each stage of the design process, which were contextual inquiry, ideation, concept development and prototype testing. Letting the care team fill in the person-centred canvas by themselves was an activity not as planned, and some students found these filled-in canvases to be helpful for their designs. The person-centred canvas played a different role when being introduced to the care team, that is, a sensitising material for designers to gain insights about PwD and their living contexts. This coincides with previous studies on design toolkit development that a tool could play different roles depending on whom it is introduced to [32]. Meanwhile, three areas for improvements were identified for the toolkit, each of which will be discussed in detail below.

To elaborate on current tools, first of all, the “Know-me” toolkit as an early-stage toolkit needs to accumulate design examples in which it is applied. The toolkits developed previously by MinD and LAUGH for designing for PwD usually provide this kind of example on their website [15,16,17,18]. The designs developed by the students in the Advanced Concept Design course were selected and added on the Know-me website as the first batch of examples to be showcased to help designers who would like to use the toolkit in the future. Next, the capability card set could adopt a layered design since some designers prefer an overview of all the capabilities while other designers would like detailed information about these capabilities. A capability card set with multiple layers could help the designers to retrieve detailed information when needed while maintaining an overview. Lastly, advice will be given to designers on how to use the toolkit with the care team. Although some students found the filled-in canvas informative, others thought the filled-in canvas contained too little information to be useful. Therefore, this type of guidance might help designers engage and gather relevant information from the care team without increasing their workload significantly.

As for the elements that were indicated by the students as worth to be added to the “Know-me” toolkit, one of them was mainly related to the lack of direct interactions with PwD due to the COVID-19 pandemic. Specifically, some students would like to receive advice on how to do remote testing with PwD. The other two elements were more generic, one was on providing more literature resources about PwD for additional background information, and the other was on how to evaluate the responses of PwD towards a proposed design. Whether these elements should be added and to what extent ought to be considered further, as the “Know-me” toolkit was designed not to be all-encompassing and should be used with other tools and resources. The “Know-me” toolkit could, however, point out some other tools and resources that might be helpful for the users.

The accessibility of the “Know-me” toolkit could be improved. The website of this toolkit should ideally be distributed more so that more designers can find this toolkit. The website should be designed in a way to make the user manual more visible. It is important for the designers to know about the existence of the user manual before they decide to use it or not. Since the toolkit was under development in the first half of the Advanced Concept Design course, it was provided to the students at a later stage. In future use, the toolkit should be introduced to designers at the beginning of the design process and perhaps be part of courses focused on designing. The toolkit is currently digital, and not all parts can be printed easily. A printer-friendly version of the toolkit will be created so that it can be printed with an A4 format by designers themselves. This will help not only designers but also other people who would like to be involved (e.g., PwD, care team, families).

### 4.2. Comparisons with State-of-the-Art Toolkits

To triangulate with the findings of the preliminary evaluation of the “Know-me” toolkit by the students, we compared the “Know-me” toolkit with similar state-of-the-art toolkits on design for dementia care. This offered another basis for us to discuss the strengths and weaknesses of the “Know-me” toolkit. Specifically, the “Know-me” toolkit was compared with the toolkits developed by the MinD project and the LAUGH project in a feature-by-feature manner (Table 3).

From Table 3, the main strengths of the “Know-me” toolkit are the explicit involvement of healthcare professionals as co-designers in the design process, the added component in Data-enabled Design, the usage of layered design in some of the tools that can interact with users in the digital format. The tools that adopted the layered design are the co-design guide and data exploration guide. Some students indicated that the capability card set would be easier to use if it had a layered design. The weaknesses of the “Know-me” toolkit are that: it is less visible on search engines (i.e., Google), and not all components of the toolkit can be printed easily. These weaknesses are also the areas for improvement of the “Know-me” toolkit in the future, which also coincide with the feedback by the students. We think the tension between “layered design” and “printer-friendliness” could be solved by developing two versions of the Know-me toolkit: one version is interactive with layered design; the other version is printer-friendly. The users can choose which version they prefer during the design process.

### 4.3. Limitations

Our study has a few limitations. First, the students involved had relatively homogenous education backgrounds leading to selection bias. In addition, the toolkit was introduced to the students at a later stage, so the students were not able to apply the toolkit in the whole design process in order to evaluate it. Moreover, the answers to the open questions were short, mostly about 1 to 2 sentences. Considering the students have other study activities to finish, the author only followed up on the answers which were ambiguous and needed further clarification for the data analysis. Not being able to interact with PwD directly during the COVID-19 pandemic was another limitation of this evaluation study. This caused some tools in the toolkit to be used less than others (e.g., co-design guide) due to a lack of direct contact with PwD; however, it is ethical to prioritise the health of PwD during the pandemic. Triangulating the feedback of the students by a comparison of the “Know-me” toolkit with similar state-of-the-art toolkits on design for dementia care, i.e., toolkits by LAUGH and MinD, helps with improving the validity and reliability of our findings.

### 4.4. Future Work

During the next evaluation round, a few research activities could be done to address some limitations mentioned above. First, designers with varied educational backgrounds and working experiences should be recruited to design personalised dementia care with the “Know-me” toolkit. Second, a few workshops could be organised along the design process to help the designers reflect on how they have used the toolkit, which might result in richer information with less recall bias. The care teams should also be involved to give their feedback on the usefulness and desired improvements of the toolkit.

The “Know-me” toolkit was initially developed for designers to co-design with healthcare professionals, and the evaluation study found that it was quick and easy to adapt the language and format of some parts of the toolkit to let the care team use it by themselves. This opens up the opportunities provided by the toolkit further: (1) What insights, if any, could be gained by the care team when using the toolkit by themselves? (2) What actions, if any, could the care team take based on these insights? (3) Can healthcare professionals be empowered by the toolkit to design for personalised dementia care? To answer these questions, a thorough evaluation of the adapted Know-me toolkit should be conducted with the care team beforehand in the future.

## 5. Conclusions

In this paper, we showcased “Know-me”, a toolkit for designing personalised dementia care. We envisaged that designers would co-design with healthcare professionals using the “Know-me” toolkit to support designing for personalised dementia care. With a proof-of-concept evaluation, we identified that “Know-me” could be adapted flexibly in a way that healthcare professionals can use some of the tools by themselves. Together with a feature-by-feature comparison of the “Know-me” toolkit with similar state-of-the-art toolkits, this preliminary study indicates that the “Know-me” toolkit is a helpful addition to the current pool of toolkits on designing for dementia care and worth to be developed and evaluated further.

## Figures and Tables

**Table 1:** Themes and sub-themes on how the “Know-me” toolkit is perceived to contribute to the design process with example quotes.

Theme	Sub-Theme	Example Quote
Understand the context and users	Provide knowledge about the end-users before field study	“Allow for you to get information about PwD without needing to be there in person.”
	Gain insights from the caregivers	“I do believe that I could have used the person-centred canvas to gain better insights from the caregivers.”
	Narrow down the design brief	“Design for dementia is an elaborate topic, so the toolkit gives you hands-on tips on where to start.”
	Understand the capabilities of PwD	“Capability cards helped me when designing my product that is multi-sensory and how to adjust it so that people with dementia can enjoy the product.”
Initiate the ideation	Understand the needs and wishes of PwD	“I think these tools are helpful during the ideation phase to understand the needs and wishes of the users better.”
	Offer a design direction	“In the first period, I didn’t know what to do; it gave me helpful instructions, like which direction I could think about (for instance, music therapy).”
Develop the concept	Use as a knowledge source	“I needed to design something the size of an image that PwD could still see. Therefore, I checked the capability cards.”
	Provide criteria for improving the design	“The capability cards helped to think of what you need to still improve the concept.”
Test the prototype	Answer some user testing questions	“For me, it could answer some of the questions that I wanted to determine in user testing.”
	Predict the reactions of PwD	“Give insight into the way the end-user handles certain things/products.”

**Table 2:** Themes and sub-themes on the desired improvement of the “Know-me” toolkit with example quotes.

Theme	Sub-Theme	Example Quote
Elaborate the tools	Provide more examples of how each tool could be used	“Maybe examples of how they are used in a design process, how they come back in a final design.”
	The capability cards could be more detailed	“I think it will be better if there are more classification of different stages and types of dementia in the cards.”
	Include how to use the toolkit with the caregivers	“The canvas would also have helped if the caregivers had time to fill them in.”
Add more elements to the toolkit	Add literature resources about dementia	“I think that’s ‘more literature resources for further explanation of dementia’ which means making this tool becomes a literature searching tool for designers to use.”
	Include methods for evaluating responses of PwD	“I used the positive response schedule by introducing an activity between the caregiver and the person with Dementia. It helped me understand, observe and mark the behaviour of both the PwD and the caregiver.”
	Include methods for remote testing	“I would like to see the reactions of PwD themselves. So maybe more technological support with cameras, etc.”
Make the toolkit more accessible	Visibility of the website	“More a practical thing; it is a bit difficult to find the website and also to find different sections of the website.”
	The user manual should be more visible	“Not really sure which manual you’re referring to.”
	Hardcopy and templates to make it more accessible	“Maybe these tools could be made into one booklet with templates so researchers could use it on the fly.”
	Provide the toolkit at an earlier stage of the design process	“It gave me inspiration. But these cards were shown to me at a late stage, so, unfortunately, I could not use them as much as I wanted.”

**Table 3:** Feature-by-feature comparison of the “Know-me” toolkit with similar state-of-the-art toolkits on design for dementia care.

Feature	“Know-Me” Toolkit	Toolkit by MinD	Toolkit by LAUGH
Target users	Designers and healthcare professionals	Designers	Designers
Components	Ergonomics in Ageing; Co-design; Data-enabled Design	Ergonomics in Ageing; Co-design;	Ergonomics in Ageing; Co-design;
Design goal	Personalised design	Generic design	Personalised design
Stages of dementia that are applicable	All stages	All stages	Moderate to late stages
Design examples on how to use the toolkit	Design examples available online now	Design examples available online	Design examples available online
Format	Cards, interactive tools, canvas	Cards, canvas	Booklet
Detailedness of instructions	Step-by-step instructions	Step-by-step instructions	Only design elements are explained
Interactivity and layered design	Some tools are interactive digitally with a layered design	None of the tools is interactive digitally or with layered design	None of the tools is interactive digitally or with layered design
Accessibility	Does not yet appear on the first page of Google search with the search term “design toolkit dementia”	Appears on the first page of Google search with the search term “design toolkit dementia”	Appears on the first page of Google search with the search term “design toolkit dementia”
Hardcopy availability	The interactive tools cannot be easily printed—is rectified	Can be easily printed	Can be easily printed
User manual availability	Available on the website	Rationale of each tool is explained on the website	No user manual
Design stages that are applicable	For the whole design process	For the whole design process	For the whole design process

## Data Availability

The data presented in this study are available on request from the corresponding author. The data are not publicly available due to ethical reasons.
